# A Kinect-Based Physiotherapy and Assessment Platform for Parkinson's Disease Patients

**DOI:** 10.1155/2016/9413642

**Published:** 2016-10-16

**Authors:** Ioannis Pachoulakis, Nikolaos Xilourgos, Nikolaos Papadopoulos, Anastasia Analyti

**Affiliations:** ^1^Department of Informatics Engineering, Technological Educational Institute of Crete, Heraklion, 71410 Crete, Greece; ^2^Institute of Computer Science, Foundation for Research and Technology-Hellas (FORTH), Vassilika Vouton, Heraklion, 70013 Crete, Greece

## Abstract

We report on a Kinect-based, augmented reality, real-time physiotherapy platform tailored to Parkinson's disease (PD) patients. The platform employs a Kinect sensor to extract real-time 3D skeletal data (joint information) from a patient facing the sensor (at 30 frames per second). In addition, a small collection of exercises practiced in traditional physiotherapy for PD patients has been implemented in the Unity 3D game engine. Each exercise employs linear or circular movement patterns and poses very light-weight processing demands on real-time computations. During an exercise, trainer instruction demonstrates correct execution and Kinect-provided 3D joint data are fed to the game engine and compared to exercise-specific control routines to assess proper posture and body control in real time. When an exercise is complete, performance metrics appropriate for that exercise are computed and displayed on screen to enable the attending physiotherapist to fine-tune the exercise to the abilities/needs of an individual patient as well as to provide performance feedback to the patient. The platform can operate in a physiotherapist's office and, following appropriate validation, in a home environment. Finally, exercises can be parameterized meaningfully, depending on the intended purpose (motor assessment versus plain exercise at home).

## 1. Introduction

Over six million people worldwide [1] suffer from Parkinson's disease (PD), a neurodegenerative condition that results from the damage of dopamine-producing neurons in an area of the brain known as substantia nigra. Dopamine acts as a mediator for transferring electrical signals (messages) and helps humans retain smooth, controlled, and purposeful movement. When a large percentage of those dopamine-producing neurons are damaged, the motor symptoms of PD appear. In addition, the meta-analysis of worldwide data [2] reveals and quantifies the rising prevalence of PD with age. At disease onset and in early stages, PD affects mostly motor function, while in more advanced stages one also suffers from cognitive, behavioral, and mental-related symptoms [3]. The four fundamental motor symptoms of the disease (tremor, rigidity, akinesia (or bradykinesia), and postural instability) are commonly referred to by the acronym TRAP [4]. These motor symptoms, which can be expressed in different degrees, can encumber and complicate daily activities and reduce the quality of life, especially as the disease progresses [5, 6]. Finally, nonmotor symptoms of the disease include cognitive impairment, sleep disturbances, depression, anxiety, psychosis, hallucinations, pain, and fatigue.

A cure for PD has not yet been discovered. However, six categories of drugs are commonly used to control PD-related symptoms [7] and maintain body functionality at reasonable levels throughout the lifetime of the patient. The active ingredients include levodopa, dopamine agonists, MAO-B inhibitors, COMT inhibitor, anticholinergic agents, and amantadine. Significant variabilities of symptoms and their severity among patients during the course of the disease make standard medication paths difficult to achieve [8]. Although levodopa is very effective at improving PD-related motor symptoms, large doses over extended periods may give rise to dyskinesia or involuntary abnormal movements, both of which further aggravate patients' walking ability and motor function. Accordingly, recent clinical practice issues agonists and postpones levodopa for later stages, when motor symptoms are not satisfactorily controlled [9]. Later/more advanced stages of the disease may require combinations of levodopa, dopamine agonists, COMT inhibitors, and MAO-B to more effectively control symptoms [8].

In parallel to medical treatment, physiotherapy has proven highly effective in controlling and delaying PD-related symptoms [10–13] and is openly supported by a number of Parkinson clinical facilities and associations. For example, the Parkinson Society of Canada [14] provides detailed online instructions on stretching and other physical exercises. Randomized controlled trials, such as [15], support that physical exercise such as stretching, aerobics, unweighted/weighed treadmill, and strength training improves motor functionality (leg stretching, muscle strength, balance, and walking) and quality of life. The “training BIG” strategy for PD rehabilitation [16], in particular, has shown especially promising results. Training BIG advocates exercises that deploy the entire body both in seated and in standing posture (such as reaching and twisting to each side or stepping and reaching forward) that are to be performed at maximum range of motion (maximum amplitude). A recent review [17] of relevant technology-aided rehabilitation platforms gleans a number of design principles that must characterize physiotherapy solutions for the PD population.

In this work we report on a Kinect-based, augmented reality, real-time physiotherapy platform tailored to PD patients. It is meant to augment and not replace physiotherapy sessions and allows a patient to exercise in front of a large TV monitor—instead of in front of a mirror—to control posture, but with added useful digital artefacts. The platform can operate in the exercise room of a physiotherapist and individual exercises can be parametrized to the abilities or physiotherapy needs of an individual patient. The ability for parametrization is very important for progressing diseases like PD, as they allow for tailoring of different exercises to patients not only in the first few establishing physiotherapy sessions, but also as medium-term gains from exercise or medication are realized or even as the disease progresses. Currently a small collection of exercises based on those commonly practiced in traditional physiotherapy for PD patients has been implemented in the platform. We plan to expand the existing exercise compendium to allow physiotherapists more freedom in shaping customized exercise schedules to individual patients.

The choice to employ the Kinect sensor is of fundamental importance in the design and implementation of the platform, because it offers a unique opportunity to create a “closed-loop” system which facilitates patient monitoring during execution of an exercise to provide real-time visual feedback, such as on-screen guiding artefacts and repetition counters to alert the patient on his/her performance. Perhaps more important to clinical motor assessment is the ability of the system to quantify patient mobility and dexterity on a per-exercise basis using exercise-specific performance metrics. Truly, such quantitative “kinesiological imprints” can be affected by various factors, such as time of day, tiredness of the patient, effectiveness of administered drugs, and on/off times. However, more meaningful and statistically sound results over a period of a few days of using the platform can be collected by controlling those variables that can be controlled: for example, exercise early in the day and at the same time after taking medication. As a result, customized, daily exercise schedules afford the possibility to collect a time series of performance data that can be usefully correlated with, for example, detailed medication history records and disease progress.

Indeed, as PD progresses over a number of years (typically around 15 after initial diagnosis), patients may show inconsistent response to dopaminergic medications, leading to shorter periods of adequately controlled symptoms (on times), more extended periods where the medication is not working sufficiently well (off times), and possibly erratic “wearing-off” transitions from on to off times. By this time, many patients exhibit more severe motor symptoms and their quality of life is seriously affected. Following an assessment of their status (motor symptoms, response to medication, on and off times, and wearing-off periods), the attending neurologist may indicate the alternative path of undergoing Deep Brain Stimulation (DBS) surgery [18]. DBS intervention may also be indicative to younger PD patients with more active life styles and work schedules if they suffer from drug-resistant tremor [19]. This minimally invasive and reversible procedure entails (a) preoperative imaging to determine the best access path to the intended target, (b) surgically implanting and deploying a multielectrode probe in strategically selected areas of the brain such as the subthalamic nucleus (STN), and (c) connecting the probe via an extension wire to a small battery-powered neurostimulator (which is later on implanted at a comfortable place under the skin). The neurostimulator regulates the signals sent to the leads via a programmable computer chip and can be parametrized and tested to effectively block brain signals that cause PD symptoms. The system remains with the patient and requires battery change every few years. Postoperative assessment includes quantifying the response of the patient to different stimulation patterns, which varies among patients due to, for example, neurobiological state and actual placement of the probe leads. By providing quantitative performance metrics on the pre-op and post-op motor abilities of a patient, our physiotherapy platform could quantify the effectiveness of each programmed DBS stimulation pattern on patient mobility. It would be then possible to select those stimulation patterns that prove most effective for the given patient.

## 2. Materials and Methods

### 2.1. Platform Specification

**Figure 1:**
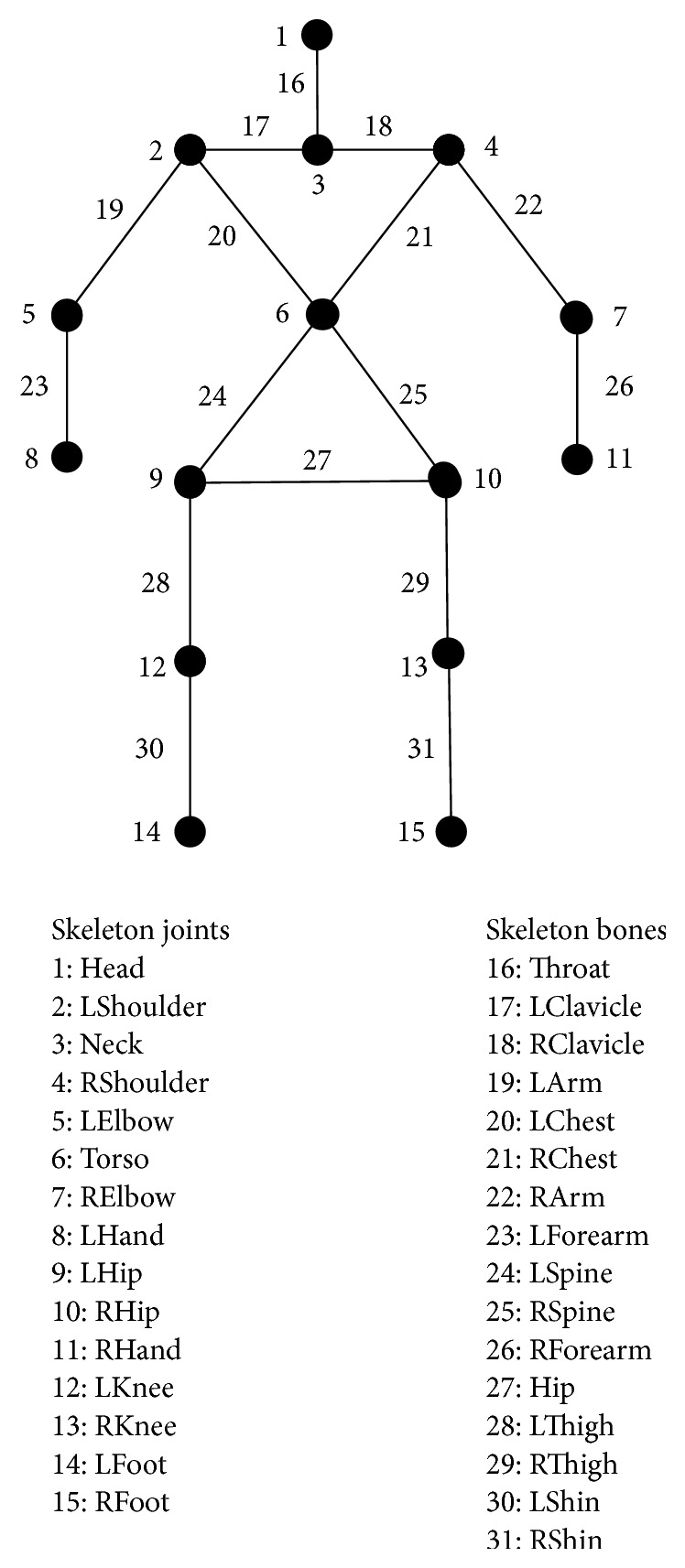
Joints utilized in the physiotherapy platform.

The physiotherapy platform combines a number of key hardware and software technologies to provide the desired functionalities. Specifically, a Microsoft Kinect v1 sensor supplies real-time 2D (RGB camera) and depth (IR depth camera) streams. These streams are processed by MS Kinect SDK v1.8 functions to (a) identify a patient in front of the sensor, (b) extract that person's skeleton as a hierarchy of nodes with 3D location data, and (c) update that skeleton in every frame to track the patient. The full skeletal model appears in Figure 1. For every frame where a player is visible and tracked, joint information includes joint's position in 3D space as well as a tag with two possible values: “tracked” for clearly visible joints or “inferred” for joints that are not clearly visible (e.g., occluded by another body part) but their position can be calculated from other (tracked) joints.

The logic for each exercise has been coded in the C# programming language in the Unity 3D v4.6 game engine (which works well with Kinect v1.0 and the MS Kinect SDK v1.8). However, while the Kinect SDK libraries are based on Microsoft's  .NET 4 framework, Unity's mono framework is based on an older  .NET framework version and a number of functions cannot be called in the same manner in the two frameworks. As a result, the Kinect SDK library cannot be directly accessed from within Unity MonoDevelop IDE. As expected, a number of custom middleware solutions appeared to alleviate that problem, that is, to expose Kinect SDK functionality inside MonoDevelop. We opted to adopt Rumen Filkov's robust KinectWrapper which was easily incorporated in our Unity project via Unity's Asset Store. The KinectWraper is essentially a customized C# script that exposes Kinect SDK (Kinect10.dll) functionality inside MonoDevelop. An additional C# script called KinectManager includes functions to read data from the Kinect sensor to build a skeleton.

In addition, synchronized RGB (camera) and depth map stream data has been combined as shown, for example, in Figure  3(C) for the first exercise, to create an experience very similar to working out in front of a mirror (a large screen TV is much more effective than computer monitor in that respect). The overlaid AR artefacts are a straightforward result obtained by projecting the selected joints of interest from 3D onto the 2D vertical plane corresponding to the image of the RGB camera.

### 2.2. Implemented Exercises Tailored to Parkinson's Disease

**Figure 2:**
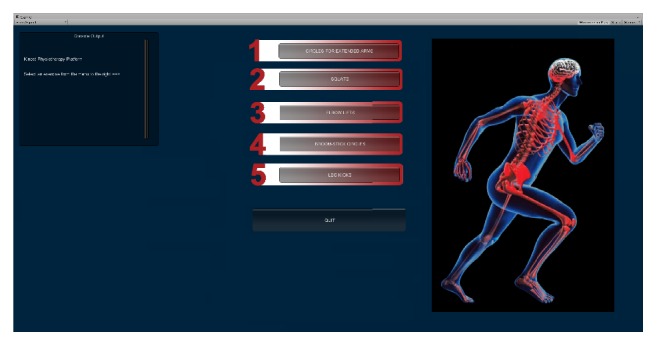
Screenshot of the platform's home screen showing the exercise menu.

Five representative exercises have been adopted from those commonly found in physiotherapy exercise curricula for PD patients, some of which can be executed from a standing position and others from a seated position to show the capabilities of the platform. The exercise menu appears in Figure 2 and includes the following five exercises: (1) circles for extended arms, (2) squats, (3) elbow lifts, (4) broom-stick circles, and (5) leg extensions/kicks. The following requirements for the selection of these exercises were used.

- The exercises must be possible to be performed reasonably well by PD patients with mild to moderate symptoms (stages 1 through 3 in the Hoehn and Yahr [20] scale, i.e., without severe postural instability/motor impairment).
- For the entire duration of an exercise, the patient's posture must adhere to the capabilities of the Kinect sensor. Practically, this means that the Kinect sensor (in reality its supporting SDK) must at all times be able to track the patient's body and successfully extract that patient's skeletal model for the full range of movements required for the exercise (e.g., a limb should not occlude another limb).

Each exercise employs either linear or circular movement patterns that pose very low processing demands on real-time computations. In addition, Kinect-provided 3D joint data are fed in real time to the game engine and compared to control routines relevant to the exercise being executed to assess proper posture and body control for the entire duration of the current repetition. Visual feedback is provided via AR artefacts which show how the skeleton is tracking the patient and repetition counters. When an exercise is complete, performance metrics appropriate for that exercise are computed and displayed on screen (a) to enable the attending physiotherapist to fine-tune the exercise to the abilities/needs of an individual patient and (b) to provide performance feedback to the patient. The exercises implemented in the current version of the platform and the performance metrics that are produced (and can be stored to establish a sequence of historical data for offline analysis) are discussed directly below.

**Figure 3:**
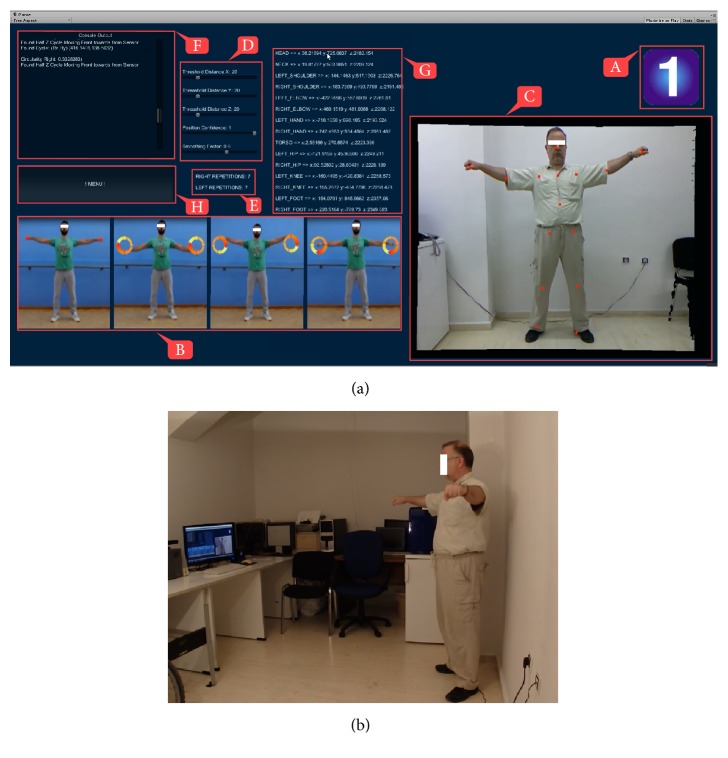
(a)* Exercise 1* is an arm stretching/strengthening exercise executed from a standing position. The included screenshot is a snapshot of the screen in its most informative mode. Several regions of information are identified as follows. Region “A” is the number id of the exercise being executed. Region “B” shows four key snapshots of a trainer performing the exercise. Region “C” is an insert of Kinect's RGB camera view into the platform, depicting an actor performing the exercise in real time in front of the sensor. The red dots superimposed on the actor are skeleton joints whose 3D coordinates are being calculated in real time and in the current frame are projected on the 2D frontal vertical plane as guiding digital artefacts. Region “D” lists three groups of variables used to minimize the effect of random joint positional errors arising from the hardware and to detect macroscopic movements accurately. Region “E” shows the number of detected repetitions for each arm (in the screenshot, the actor has completed seven repetitions for each arm and is working on the next repetition). Regions “F” and “G” show debugging information that is useful to the developers to effectively fine-tune the parameters in region “D”. Finally, the menu button in region “H” takes us back to the main menu shown in Figure 2. It is worth mentioning that the regions visible to the patient in normal (nondebugging) operation are A, B, C, E, and H. (b) A snapshot of an agent performing Exercise 1 in front of a 25′′ monitor at a distance of approximately 2.5 m from the Kinect sensor (placed to the left of the monitor). In actual deployment, a much larger 55–58′′ monitor would be preferred.

*Exercise 1*. Facing the Kinect sensor, the patient assumes a relaxed standing stance with feet spaced apart at about shoulder width and with both arms extended laterally and in parallel to the transverse axis, as shown in the first snapshot of Figure  3(B). Then, from that stance, he/she has to complete *N* cyclic movements of the wrists where both extended arms move in unison, as shown in the sequence of snapshots in Figure  3(B). The default value of *N* for each exercise is 10. During each such cyclic movement, the arms must remain extended laterally while the wrists describe circles on imaginary planes that are parallel to the sagittal plane. Game code relevant to this exercise checks for correct execution as follows:

- Ideally, each arm must remain fully extended laterally for the duration of the exercise. Deviations from a fully extended arm pattern are calculated from the 3D coordinates of the (detected) shoulder, elbow, and wrist joints for that arm, so that the shoulder-elbow-wrist opening angle is computed in real time.
- The motion pattern of each wrist projected to the sagittal plane is checked for circularity, meaning it must follow a superior-anterior-inferior-posterior-superior sequence (or, alternatively, a superior-posterior-inferior-anterior-and-back-to-superior sequence). This check is meant to count only circular patterns and not linear patterns, such as the wrist moving vertically, horizontally, or even along a diagonal.

A repetition is considered successful if it passes both tests described directly above, in which case an appropriate on-screen counter (*L*_*s*_ for the left arm or *R*_*s*_ for the right arm) is incremented by one, as shown in Figure  3(E). On the other hand, a repetition (for the left or right arm) is considered a failed repetition if the corresponding wrist prescribes at least half a circle but does not complete that circle, in which event a “failure” counter (*L*_*f*_ or *R*_*f*_) is incremented accordingly. When *L*_*s*_ or *R*_*s*_ reaches *N*, the success and failure counters corresponding to that arm stop incrementing. The exercise is considered complete when both *L*_*s*_ and *R*_*s*_ equal *N*, at which point the following performance metrics are shown on screen, separately for each arm: (a) the total of number of failed repetitions *L*_*f*_ or *R*_*f*_ and (b) a circularity metric showing the ratio of the average superior-to-inferior distance divided by the anterior-to-posterior distance. Clearly, for a perfectly executed exercise, *L*_*f*_ = *R*_*f*_ = 0 and circularity = 1.

These metrics lead to direct interpretation (a key design requirement for this collection of exercises). For example, large departures of both *L*_*f*_ and *R*_*f*_ from zero may mean that the patient has not understood the exercise or that the exercise is too hard for him/her. Alternatively, consistently disparate values for *L*_*f*_ and *R*_*f*_ (e.g., *L*_*f*_ close to zero but *R*_*f*_ significantly higher) may reveal a measurable differential in mobility control between the left and right sides. Finally, circularity metric values that deviate significantly from 1 show that the patient favors vertical or horizontal elliptical patterns for that arm. It is then up to the physiotherapist to parameterize the exercise depending on the priorities set forth for a given patient as well as the capabilities of that patient. To quantitatively assess the motor function of a patient in the context of the present exercise (but also for any other exercise in the current compendium), one would explore the parameter space to “push” the patient near the limits of his/her abilities and obtain more valid results over a period of sessions. That would also be the suggested approach to establish as accurate as possible baseline of patient's motor abilities in the period before Deep Brain Stimulation (DBS) surgery, but also in the following months to assess the effectiveness of each stimulation pattern. On the other hand, parameterization of the platform for home-based use should probably aim at encouraging patients to exercise more by posing less stringent demands than in the above situations, lest they become discouraged and cease to exercise.

**Figure 4:**
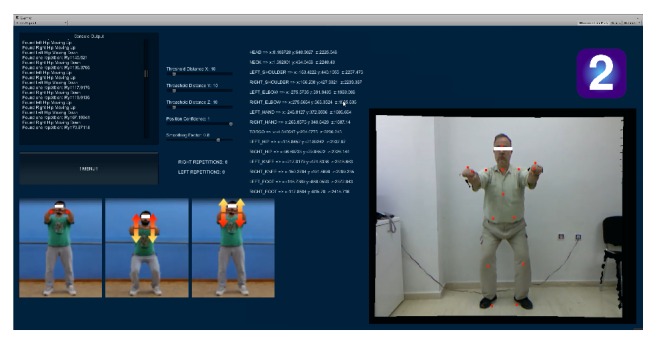
*Exercise 2*. A leg-strengthening exercise (squats) executed from a standing position. The actor shown on the right has just completed repetition 8. All other on-screen information is documented in the caption of Figure 3.

*Exercise 2*. Facing the Kinect sensor and in a relaxed standing stance (as shown in Figure 4), the patient extends both arms fully to the front (i.e., parallel to the sagittal axis). Starting from that stance, he/she has to complete a number of *N* squats. The depth of a squat is computed as the maximal distance *D* travelled vertically by the mid-hip joint. Game code relevant to this exercise checks for correct execution by counting only squats that are sufficiently deep; that is, *D* > *D*_min_, where *D*_min_ is an exercise-specific parameter set to define the difficulty of the exercise. The default value for *D*_min_ is (*L*_Thigh_ + *R*_Thigh_)/6 (see Figure 1 for the definitions of *L*_Thigh_ and *R*_Thigh_), which corresponds to an average level of difficulty. For each successful squat an appropriate on-screen counter *M*_*s*_ is incremented by one, while each failed squat increments a “failed rep” counter *M*_*f*_. The exercise completes when *M*_*s*_ reaches *N*, at which point the following two performance metrics are computed and shown on screen: (a) the number of failed squats *M*_*f*_ and (b) the average depth of a squat 〈*D*〉 as a percentage of *D*_min_ (i.e., 〈*D*〉/*D*_min_ *∗*100%).

These metrics can be used by the physiotherapist to fine-tune the exercise (number of repetitions and squat depth) to the individual patient. For example, a performance result of *M*_*f*_ = 0 and average squat depth close to or even greater than 100% (overachieving) implies that the exercise is too easy for that patient and could possibly be made harder by increasing *D*_min_. Alternatively, a larger value of Mf or one that is comparable to *N* combined with an average squat depth very close to 100% may imply that the patient has trouble performing squats this deep and the exercise must be made easier by decreasing *D*_min_.

**Figure 5:**
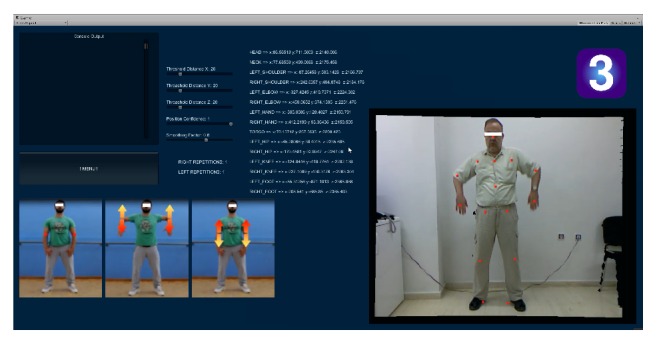
*Exercise 3*. An arm stretching/strengthening exercise (elbow lifts) executed from a standing position. The actor shown on the right has just completed repetition 1. All other on-screen information is documented in the caption of Figure 3.

*Exercise 3*. Facing the Kinect sensor, the patient assumes a standing stance with both arms relaxed and to the side as shown in the first trainer snapshot in Figure 5. From that stance, he/she has to complete *N* repetitions of slowly and purposefully lifting both upper arms in unison to a horizontal position (so that the upper arms end up almost parallel to the transverse axis at approximately shoulder height), followed by a controlled reverse movement downwards to the relaxed state. During execution of this exercise it is important to maintain (a) proper control (e.g., not to let the forearms drop under their own weight) and (b) proper posture by keeping the forearms as vertical as possible during the entire range of motion. Game code relevant to this exercise checks for correct execution in the following sense.

(i) Forearms remain close to the vertical for the duration of the exercise. Deviations from this posture are calculated in real time from the 3D coordinates of the elbow and wrist joints for each forearm. In addition, the exercise is parameterized so that this requirement can be somewhat relaxed to allow for patients that cannot attain and/or retain the required posture to still practice.
(ii) For each repetition, the vertical distances *H*_*L*_/*H*_*R*_ that must be travelled by the left/right elbow, respectively, must be close to the length of the forearm (variables *L*_Arm_/*R*_Arm_ in Figure 1) in order to ensure full range of motion. The ratios *H*_*L*_/*L*_Arm_ and *H*_*R*_/*R*_Arm_ define the difficulty of the exercise. For PD patients, ratio values close to 1 may be hard to attain and tiring to maintain over many repetitions, whereas ratio values close to, say, 0.7 pose more realistic expectations. In any case, the difficulty of the exercise can be set separately for each arm by the attending physiotherapist on a per-patient basis.

A repetition is considered successful if it passes both tests described directly above, in which case an appropriate on-screen counter (*L*_*s*_ for the left arm or *R*_*s*_ for the right arm) is incremented by one. Otherwise the repetition (for the left or right arm) is considered failed and a “failure” counter (*L*_*f*_ or *R*_*f*_) is incremented accordingly. When *L*_*s*_ or *R*_*s*_ reaches *N*, the success and failure counters corresponding to that arm stop incrementing. The exercise is considered complete when both *L*_*s*_ and *R*_*s*_ equal *N*, at which point the following performance metrics are shown on screen, separately for each arm: (a) the number of failed repetitions *L*_*f*_ or *R*_*f*_ and (b) the average maximum height 〈*H*_*L*_〉 or 〈*H*_*R*_〉 over all successful repetitions for the left or right elbow, respectively, expressed as a percentage of the upper arm length, that is, 〈*H*_*L*_〉/*L*_Arm_ *∗*100% for the left arm and 〈*H*_*R*_〉/*R*_Arm_ *∗*100% for the right arm. A perfectly executed exercise should yield *L*_*f*_ = *R*_*f*_ = 0 and an average elbow height that is close to or exceeds the value corresponding to the difficulty level set by the attending physiotherapist. These metrics can be used as follows. Large departures of both *L*_*f*_ and *R*_*f*_ from zero may mean that the exercise is too hard for that patient, in which case an attending physiotherapist may select to ease the difficulty level and/or decrease the number of required repetitions to complete the exercise. Alternatively, consistently disparate values for *L*_*f*_ and *R*_*f*_ (e.g., *L*_*f*_ close to zero but *R*_*f*_ significantly higher) reveal a measurable differential in mobility control between the left and right sides.

**Figure 6:**
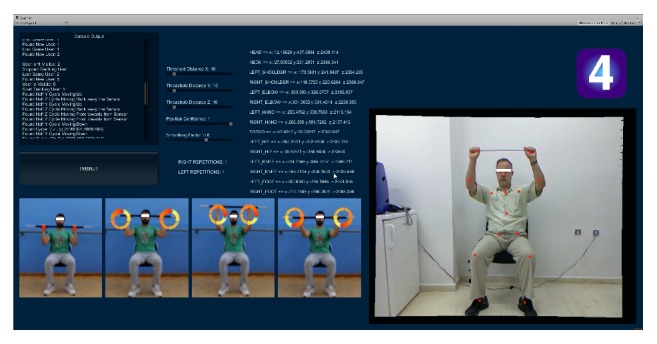
*Exercise 4*. An arm stretching/strengthening exercise executed from a seated position with the help of a short rod or light broomstick to facilitate coordinated movement of the left and right arms. The actor shown on the right has just completed repetition 1. All other on-screen information is documented in the caption of Figure 3.

*Exercise 4*. Facing the Kinect sensor, the seated patient holds a light stick such as a broomstick (which helps coordinate the movements of the left and right arms) with both hands at the initial upright stance where the wrist joints are located slightly higher than the shoulder joints (as shown in the first trainer snapshot in Figure 6). Then, from that stance, he/she has to complete *N* cyclic movements of the wrists where both arms move in unison and in phase to each other. During each such cyclic movement, the wrists describe circles on imaginary planes that are parallel to the sagittal plane. Game code relevant to this exercise checks for correct execution, in the sense that both wrists must follow a superior-anterior-inferior-posterior-superior sequence or, alternatively, both wrists must follow a superior-posterior-inferior-anterior-superior sequence. This check helps avoid linear patterns, such as vertical or horizontal wrist joint movements. The counters and metrics for this exercise are identical to those in Exercise 1.

**Figure 7:**
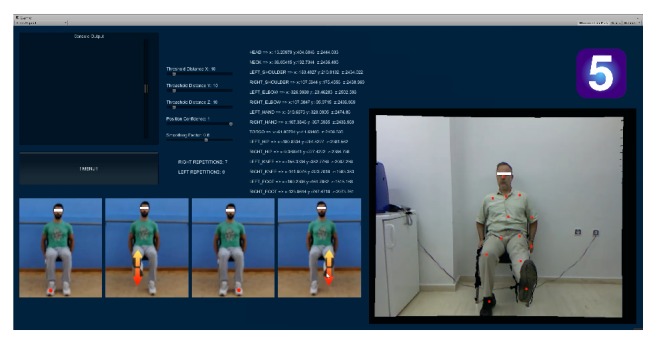
*Exercise 5*. A leg stretching/strengthening exercise (leg extensions or “kicks”) executed from a seated position. The actor shown on the right has completed 7 repetitions for the right leg and his 8th repetition for the left leg. All other on-screen information is documented in the caption of Figure 3.

*Exercise 5*. In this, final, exercise, the patient is seated facing the Kinect sensor, with feet securely planted on the ground and holding the seat of the chair with both palms for additional support (as shown in the first trainer snapshot in Figure 7). The exercise calls for the completion of *N* controlled full extensions for each leg in any order. Game code relevant to this exercise checks for correct execution, so that a repetition is considered successful if an ankle joint is lifted to a height that is above a threshold value *H*_min_ (which has a convenient default value of 0.75*∗*(*L*_Shin_ + *R*_Shin_)/2—the parameters *L*_Shin_ and *R*_Shin_ which are defined in Figure 1), which corresponds to an average level of difficulty.

For each successful extension an appropriate on-screen counter (*L*_*s*_ for the left leg or *R*_*s*_ for the right leg) is incremented by one, as shown in Figure 7. On the other hand, each failed repetition (for the left or right leg) increments the corresponding “failure” counter (*L*_*f*_ or *R*_*f*_). When *L*_*s*_ or *R*_*s*_ reaches *N*, the success and failure counters corresponding to that leg stop incrementing. Finally, the exercise is considered complete when both *L*_*s*_ and *R*_*s*_ equal *N*, at which point the following performance metrics are shown on screen, separately for each leg: (a) the total of number of failed repetitions *L*_*f*_ or *R*_*f*_ and (b) the average maximum ankle height 〈*H*_*L*_〉 or 〈*H*_*R*_〉 over all successful repetitions for the leg in question, expressed as a percentage of shin length, that is, 〈*H*_*L*_〉/*L*_Shin_ *∗*100% for the left leg and 〈*H*_*R*_〉/*R*_Shin_ *∗*100% for the right leg. A perfectly executed exercise should yield *L*_*f*_ = *R*_*f*_ = 0 and an average maximum ankle height that is close to or exceeds the value corresponding to the difficulty level set by the attending physiotherapist. Parameterization of this exercise is the same as in Exercise 1.

## 3. Discussion and Future Work

Patients with neurological disorders are known to benefit from physical practice, which improves mobility and functional independence through increased muscular strength, flexibility, and balance control. The present work reports on a Kinect-based, augmented reality, real-time assessment physiotherapy platform tailored to Parkinson's disease (PD) patients with mild to moderate symptoms (stages 1 through 3 in the Hoehn and Yahr [20] scale, i.e., without severe postural instability and motor impairment).

Main platform characteristics are as follows.

- The platform offers a persuasive augmented reality experience (by using a large TV monitor instead of a computer screen, patients are afforded the experience of working out in front of a mirror) and one that is enriched with important digital artefacts (relevant skeleton joints are overlaid on the actual image of the patient for the duration of an exercise) and feedback information (e.g., repetition counters).
- The platform is adaptable to the abilities/exercise needs of an individual patient: each exercise is parametrized to a difficulty that can be fine-tuned and tailored to each patient separately. Adaptability is important in the first few establishing physiotherapy sessions and also as medium-term gains from exercise are realized or even as the disease progresses.
- The platform has a sufficiently small footprint to operate in the office of a physiotherapist, as it requires the following hardware components: (i) an entry-level laptop such as an Intel Core i3 based machine with entry-level graphics card to run the software (around 400 USD), which is largely a direct outcome of our design decisions to use the Unity game engine and to employ light-weight processes for each exercise, (ii) the Kinect sensor (around 100 USD), and (iii) a large flat panel TV monitor (or projector) that should be placed so that patients can comfortably afford a full view of themselves in both standing and seated positions at a distance of approximately 2-3 meters from the TV/Kinect sensor location (350–400 USD for a 55–58′′ TV monitor). Even though at a distance of 2-3 meters a more modest 42′′ TV set may seem adequate, we do feel that a larger 55–58′′ set would be more satisfying, at least to more elderly patients. Finally, for a home setting, the hardware acquisition cost could almost be cut in half, considering that most living rooms are already equipped with a large TV set.

In the immediate future and in collaboration with physiotherapists we plan to validate the platform with PD patients to address safety issues and fine-tune parameters related to exercise posture and pace. At the same time, we are actively augmenting the platform with exercises among those most commonly practiced in traditional PD physiotherapy to more effectively enrich customized physiotherapy schedules on a per-patient basis. The next step would be to seek funding and partners to make the platform available to a large base of physiotherapists and also PD patients who are willing to run it in their homes. In addition to affording daily customized exercise schedule to a PD patient, it will then be possible to collect a time series of performance data that can be usefully correlated with, for example, detailed medication history records and disease progress.

## References

[1] EPDA http://www.epda.eu.com/en/.

[2] Pringsheim T., Jette N., Frolkis A., Steeves T. D. L. (2014). The prevalence of Parkinson's disease: a systematic review and meta-analysis. *Movement Disorders*.

[3] Chaudhuri K. R., Healy D. G., Schapira A. H. V. (2006). Non-motor symptoms of Parkinson's disease: diagnosis and management. *Lancet Neurology*.

[4] Jankovic J. (2008). Parkinson's disease: clinical features and diagnosis. *Journal of Neurology, Neurosurgery and Psychiatry*.

[5] Opara J. A., Brola W., Leonardi M., Błaszczyk B. (2012). Quality of life in Parkinson's disease. *Journal of Medicine and Life*.

[6] Den Oudsten B. L., Van Heck G. L., De Vries J. (2007). Quality of life and related concepts in Parkinson's disease: a systematic review. *Movement Disorders*.

[7] American Parkinson Disease Association http://www.apdaparkinson.org/.

[8] Horstink M., Tolosa E., Bonuccelli U. (2006). Review of the therapeutic management of Parkinson's disease. Report of a joint task force of the European Federation of Neurological Societies and the Movement Disorder Society-European Section. Part I: early (uncomplicated) Parkinson's disease. *European Journal of Neurology*.

[9] Semenchuk M. R. (2012). *Medical Management of Parkinson's Disease*.

[10] Rodrigues-de-Paula F., Oliveira Lima L. (2010). Physical therapy—exercise and Parkinson's disease. *International Encyclopedia of Rehabilitation*.

[11] Ebersbach G. (2014). Rehabilitative therapy in patients with Parkinson's disease. *Basal Ganglia*.

[12] Dibble L. E., Addison O., Papa E. (2009). The effects of exercise on balance in persons with parkinson's disease: a systematic review across the disability spectrum. *Journal of Neurologic Physical Therapy*.

[13] Redecker C., Bilsing A., Csoti I. (2014). Physiotherapy in Parkinson's disease patients: recommendations for clinical practice. *Basal Ganglia*.

[14] Exercises for people with Parkinson's, http://www.parkinson.ca/atf/cf/%7B9ebd08a9-7886-4b2d-a1c4-a131e7096bf8%7D/EXERCISEMAR2012_EN.PDF

[15] Goodwin V. A., Richards S. H., Taylor R. S., Taylor A. H., Campbell J. L. (2008). The effectiveness of exercise interventions for people with Parkinson's disease: a systematic review and meta-analysis. *Movement Disorders*.

[16] Farley B. G., Koshland G. F. (2005). Training BIG to move faster: the application of the speed-amplitude relation as a rehabilitation strategy for people with Parkinson's disease. *Experimental Brain Research*.

[17] Pachoulakis I., Papadopoulos N., Spanaki C. (2015). Parkinson’s disease patient rehabilitation using gaming platforms: lessons learnt. *International Journal of Biomedical Engineering and Science*.

[18] Benabid A. L., Chabardes S., Mitrofanis J., Pollak P. (2009). Deep brain stimulation of the subthalamic nucleus for the treatment of Parkinson's disease. *The Lancet Neurology*.

[19] Deli G., Balás I., Dóczi T. (2015). Deep brain stimulation can preserve working status in Parkinson's disease. *Parkinson's Disease*.

[20] Goetz C. G., Poewe W., Rascol O. (2004). Movement disorder society task force report on the Hoehn and Yahr staging scale: status and recommendations The Movement Disorder Society Task Force on rating scales for Parkinson’s disease. *Movement Disorders*.

